# Using Bran of Ancient and Old Grains for Wheat Bread Production

**DOI:** 10.3390/foods14050860

**Published:** 2025-03-03

**Authors:** Oumayma Toumi, Costantino Fadda, Alessandra Del Caro, Paola Conte

**Affiliations:** Department of Agricultural Sciences, University of Sassari, Viale Italia, 39/A, 07100 Sassari, Italy; t.oumayma@studenti.uniss.it (O.T.); delcaro@uniss.it (A.D.C.); pconte@uniss.it (P.C.)

**Keywords:** ancient grains, bran, emmer, einkorn, landraces, sensory evaluation

## Abstract

In the current era of heightened awareness regarding the impact of food choices, there has been a noticeable shift towards revisiting traditional ingredients. Following the growing interest in ancient grains, this study evaluated their potential use for enriching modern wheat dough and bread. The effects of substituting 20% of wheat flour with the bran of seven ancient grains on dough’s rheological properties and bread quality were assessed. The bran-enriched doughs maintained high stability (ST) values and showed an enhanced elastic behavior compared to the control. Nonetheless, a reduction in dough extensibility (E) was also noted. In terms of bread measurements, all bran-enriched breads exhibited a lower specific volume and a darker crust and crumb compared to the control bread. However, not all of the bran breads showed a harder and chewier loaf texture. The composite breads also exhibited enhanced total dietary fiber (TDF) and polyphenol content. A sensory evaluation revealed that Garfagnana (GAR) and Norberto (NOR) bran-breads received the highest overall liking scores. In conclusion, the incorporation of ancient grain brans presents a promising approach to enhancing modern wheat doughs and breads, offering nutritional benefits without significantly compromising their sensory and textural properties.

## 1. Introduction

Since prehistoric times, wheat (*Triticum* spp.) has continued to be one of the most consumed crops worldwide, with a global production estimated at 811 million tons cultivated on around 219 million hectares in 2022 [1]. At present, the most widely grown species of wheat in the world are common wheat (*Triticum aestivum* L.), also known as “bread” wheat (accounting about 95% of global annual production), followed by durum wheat (*Triticum turgidum* L. var. *durum* Desf.), usually referred to as “pasta” wheat [2]. These modern wheats are the product of the early domestication that took place in the Fertile Crescent of the Middle East thousands of years ago through genetic selection and cross-breeding programs [3]. In effect, wild wheat progenitors were morphologically and physiologically modified by the selection of advantageous genetic traits to obtain improved varieties that meet human needs and are better adapted to agricultural practices [4]. These changes were referred to as “domestication syndrome” and mainly involved conversion from hulled forms (where the glumes of the flower adhere tightly to the grain and are not removed by threshing) to free-threshing forms (where the naked grain is released upon threshing), as well as the loss of spike shattering at maturity and the increase in seed size [5]. Although these selections facilitated grain processing and led to the development of high-yielding cultivars, their intensity caused a significant loss of genetic diversity among cultivated wheat genotypes [6]. Furthermore, modern breeding is assumed to be the root cause of the “yield dilution phenomenon”, leading to lower concentrations of minerals in modern wheat grains [7]. Consequently, the essential need for crop diversification combined with the increasing demand for a balanced healthy food system generated a revived interest in rediscovering ancient wheat grains. Other compelling arguments strengthening this trend include the low-input farming systems, adaptability to climate change, and the higher yield potentials of marginal lands and the organic farming of ancient wheat cultivars [8]. These factors can also be appreciated for their contribution to regenerative agricultural practices. Moreover, ancient grains are considered superior to modern ones in terms of micronutrient content and antioxidant compounds [9]. It is important to note that this perceived superiority requires further investigation, as the available studies were limited to a restricted range of genotypes of ancient and modern wheat species, without the ability to fully account for environmental effects [2,7].

The definition of ancient wheats is ambiguous and has divided the scientific world. Some research studies relied on the literal meaning, defining ancient wheats strictly as ancestral or historically old wheat species [10]. Others followed a definition based on historical dates and events, taking the green revolution as a watershed and classifying them as those belonging to the genus *Triticum* that originated prior to 1961 and were not subjected to intensive genetic improvement programs [10]. In addition, the presence in the literature of terms such as old wheat varieties and landraces led to greater confusion. In the present study, the following classification was adopted: ancient wheats, as defined by Giambanelli et al. [11], are “populations of primitive grains, which were not subject to any modern breeding or selection, and which retained characters of wild ancestors, such as large individual variability, ear height, brittle rachis, and low harvest index”. Einkorn (*Triticum monococcum* ssp. *monococcum*), emmer (*Triticum turgidum* ssp. *dicoccum*), Khorasan (Oriental wheat) (*Triticum turgidum* ssp. *turanicum*), and spelt (*Triticum aestivum* subsp. *spelt*) are the most widely known ancient species that were cultivated historically, but these are only grown in limited areas within organic or traditional low-input farming at present. Old wheats and landraces, on the other hand, represent emmer and durum wheat cultivars generated by natural and farmers’ selection that were exclusively cultivated up to the middle of the 20th century until they were subsequently replaced by modern cultivars after the green revolution [5,12]. In order to leverage ancient wheat qualities, active breeding programs were recently developed. A group of improved varieties has been developed, characterized by enhanced agronomic and qualitative traits while maintaining the functional properties of ancient grains [13]. In Italy, modern improved genotypes were developed through selections and crossing, such as the Monlis and Norberto einkorn genotypes selected from landraces and the local population (listed in the einkorn section of the Italian register in 2006 and 2017, respectively) and the Giovanni Paolo and Padre Pio emmer genotypes obtained by crossing cultivated emmer and durum wheat cultivars (registered in the emmer section in 2008 and 2016, respectively) [14]. These cultivars have also been found to have an adequate protein composition and technological properties that enable them and their products (i.e., wheat, germ, and bran) to be used for functional bakery products and the pasta industry [15,16].

Bran is a wheat milling by-product forming 14 to 19% of the whole wheat grain. Depending on the milling process, different bran-rich streams can be identified based on their particle size distribution and endosperm content, such as regular bran (coarse bran), fine bran (coarse weatings), and middlings (fine weatings) [17]. Generally, the constituents of regular wheat bran layers include pericarp (6% to 23%), seed coat and nucellar epidermis (6% to 30%), aleurone layer (33% to 52%), and starchy endosperm (9% to 35%) [18,19,20]. Several studies have analyzed the chemical composition of this by-product, revealing that it is predominantly composed of nonstarch carbohydrates, including arabinoxylan, cellulose, fructan, and mixed-linkage β-D-glucan [21,22,23,24], and thus its dietary fiber content ranges between 35 and 50%. In addition to these main components, other constituents have been identified as integral to the composition of bran. For instance, wheat bran has been shown to be a relevant source of protein (12% to 26%), lipids (3% to 4%), lignin (3% to 10%), minerals (5% to 7%), phytic acid (4.5% to 5.5%), and phenolic acids (0.4% to 0.8%) [20,21,22,25]. The percentage of these constituents is closely linked to the genetic characteristics of the wheat variety, as well as the environmental factors that may influence it. Wheat bran’s rich nutritional profile positions it as a suitable candidate for food product fortification. Epidemiological studies have shown that the consumption of bran-rich products offers physiological benefits due to its high levels of total dietary fiber (TDF), as recognized by the European Food Safety Authority (EFSA) [26]. In the last decade, the incorporation of wheat bran into cereal-based foods such as cakes [27], biscuits [28], pasta [29,30], and bread [31,32,33] has been extensively explored. These studies have demonstrated that the positive effects of wheat bran addition on the sensory properties and product nutritional quality are directly associated with the level of enrichment. In the case of bread, the level of wheat bran addition varied between 0% and 20% [31], 8.9% and 9% [32], and 7% and 25% [33]. The previously mentioned studies consistently demonstrated an increase in TDF with higher enrichment levels, accompanied by a concurrent reduction in specific volume and crumb luminosity. Consequently, the review of the available literature suggests that a 20% enrichment level could be considered a reliable percentage to maximize the nutritional benefits of wheat bran and optimize the fiber content in bran-rich bread.

Therefore, the aim of this study is to evaluate the potential use of bran from ancient grains for the enrichment of common wheat doughs and breads. The rheological properties of dough and the bread quality of common wheat flour substituted with 20% of bran from each studied cultivar were assessed. The resulting functional breads were evaluated to identify the best final product in terms of technological, nutritional, and sensory qualities.

## 2. Materials and Methods

### 2.1. Raw Materials

The seven wheat genotypes evaluated in this study were grown during the 2016/2017 and 2017/2018 growing seasons at the experimental farm of the University of Sassari located in Ottava (Sardinia, Italy, 41° N; 8° E; 80 m above sea level) (Table 1). The harvested wheat of each genotype (a mix from both growing seasons) underwent milling processing in an external university laboratory. Flours were collected for other purposes and the resulting brans were ground through a Lab Mill 3100 (PerkinElmer, Waltham, MA, USA) equipped with a 0.5 mm outlet grid to obtain superfine treatments. The used flour is a common wheat (*Triticum aestivum*) type 00 supplied by SIMEC Spa (Santa Giusta, OR, Italy) with the following chemical composition: 14% moisture, 11.6% protein, 3.8% fibers, 1.5% lipids, and 0.1% ash. Fresh compressed yeast and sodium chloride were acquired from a local supermarket.

### 2.2. Proximate Chemical Composition of the Studied Wheat Brans

Moisture, total lipids, and ash content were determined according to the AACC Approved Methods of Analyses [34]. Protein content was calculated using the Kjeldahl method (N × 6.25) and total dietary fibers (TDF) were calculated using the AOAC method 985.29 [35], using the K-TDFR 200A analytical kit (Megazyme International Ireland Ltd., Wicklow, Ireland). Analyses were conducted of the bran of the seven wheat cultivars (as raw material), as well as their respective bran-rich breads, in triplicate.

### 2.3. Particle Size Distribution

The particle size distribution of the different bran samples was determined using the sieve analysis method ASTM C136-06 [36]. A 250 g aliquot of bran, which was thoroughly mixed to ensure homogeneity, was sieved through a set of sieves with mesh sizes of 510, 450, 400, 350, 300, 200, and 100 µm. Proper sifting was achieved using a Vibratory Sieve Shaker (Verder Scientific, Aartselaar, Belgium), which shook the sieves for 30 min at a frequency of 1.5. The weight of the material on top of each sieve was measured and used to calculate the cumulative particle size distribution of the sample. The analysis was performed in triplicate.

### 2.4. Water Retention Capacity

Water retention capacity (WRC) was measured through applying the traditional centrifugation method, following the AACC International Method 56-11-02 [37]. In falcon tubes, a 1 g sample of wheat bran from each cultivar was soaked in 10 mL of deionized water for 60 min. Subsequently, all falcon tubes were centrifuged for 10 min (2680× *g*). After discarding the supernatant from the pellet, falcon tubes were tilted at an angle of 45° for 15 min in order to drain the samples. The residue was weighed, and the WRC was calculated by subtracting the initial sample mass. The analysis was conducted in triplicate.

### 2.5. Dough Mixing Properties

The mixing properties of composite doughs, prepared with 80% common wheat flour and 20% of the studied ancient wheat brans, and control dough, composed of 100% wheat flour, were analyzed using a Farinograph-TS (model 827507, Brabender, Duisburg, Germany) equipped with a mixer S 300 N, according to the standard procedure AACC 54-21.01 [38]. The following farinograph parameters were evaluated: water absorption (WA, %, amount of water required to reach a consistency of 500 ± 20 Brabender Units (BU)), dough development time (DDT, min time to attain maximum consistency), dough stability, (ST, min, time to maintain the maximum dough consistency) and dough-softening (DS, BU, the difference between maximum consistency and the center line of the mixing curve after 12 min, starting from DDT). Analyses were conducted in duplicate.

### 2.6. Dough Preparation and Breadmaking Procedure

The straight dough method, consisting of mixing all the ingredients in one step, was used. After being solubilized in aliquots of warm water (26 °C), 2% compressed yeast and 1.8% NaCl (flour weight basis) were mixed, along with wheat flour and a 20% substitution (of the total flour content) with bran from each genotype. The same formulation was used for the control sample without the addition of bran. The optimal water quantity for each formulation was previously determined according to the Farinograph-TS (model 827507, Brabender, Duisburg, Germany). The mixing process was conducted using a mixer (specifically, a Kitchen Aid Professional, Model 5KSM7990, St. Joseph, MI, USA) equipped with a stainless-steel dough hook set to speed 2 for a total mixing time of 10 min. The formed bulks were fermented in a climate chamber (Tecnomac Lev2+, Castel MAC Srl Veneto, Italy) for 30 min at 30 °C and 95% relative humidity (RH), then molded into even dough pieces of 250 g to fit in the rectangular baking pans, proofed again to double up their initial volume (30 °C; 95% RH), and finally baked in a convection oven (Europa, Malo, VI, Italy) at 200 °C for 40 min. A total of three loaves of bread were produced for each formulation and production batch (*n* = 2). All bread measurement analyses were conducted after the cooling time (4 h).

### 2.7. Dough Measurements

#### 2.7.1. Dough Viscoelastic Properties

Frequency sweep tests were conducted using a rotational rheometer (MCR-92, Anton Paar GmbH, Inc., Graz, Austria) assisted by a Peltier temperature-control system and a parallel plate geometry (50 mm diameter, 1 mm gap). Dough samples were prepared without the addition of yeast to prevent interference from gas bubble development. The resulting doughs were left to rest for 20 min before taking the rheological measurements at a controlled temperature (20 °C). Small deformation shear oscillatory tests were performed within the linear viscoelastic region (previously identified through a preliminary strain sweep test at a constant frequency of 10 Hz and a strain varying from 0.001 to 100 s) in the range of 0.1–10 Hz at constant strain (0.01%). As a function of frequency, the parameters of storage modulus (G′, Pa), loss modulus (G″, Pa), and loss tangent (tan δ) were measured. For each formulation, two repetitions per batch were analyzed.

#### 2.7.2. Dough Extensibility Measurements

The uniaxial extensional properties of doughs were determined using a TA-XT plus texture analyzer (Stable Micro Systems, Surrey, UK) equipped with the Kieffer dough extensibility rig and a 30 kg load cell. Small dough pieces of 50 g (prepared by mixing water, 80% flour, and 20% bran) were placed in a Teflon mold with a grooved base, which was then used to press dough pieces and form homogenized dough strips. The sample-containing press was kept in a climate chamber at 30 °C and 90% RH for 40 min to prepare the strips for measurements. The analysis was conducted on a minimum of ten dough strips per batch (*n* = 2) under the following conditions: pre-test speed of 2.0 mm/s, test speed of 3.3 mm/s, post-test speed of 10.0 mm/s, trigger force of 5 g, and an extensibility distance of 100 mm. Resistance to extension (N), also referred to as the maximum peak force, and extensibility (mm), defined by the distance required to break the dough strips, were evaluated from the registered force–distance curves.

#### 2.7.3. Rheofermentometer Analysis

The evaluation of dough behavior during fermentation was assessed through a Rheofermentometer F3 (Chopin, Villeneuve-La Garenne, France) that is able to register dough development and both carbon dioxide production and retention. Doughs were prepared as previously described in the breadmaking section. A total of 315 g of each functional dough sample was laid in the apparatus fermentation chamber then coated with 2000 g cylindrical weight equipped with an optical sensor for a duration of 180 min at 28.5 °C. The acquired parameters from the dough development curve were the height of maximum dough development (Hm, mm), the time required to obtain the maximum dough rise (T1, min), the dough height at the end of the test (h, mm), the decrease in the dough volume at the end of the test ((Hm-H)/Hm), %), and the time at which the dough begins to collapse after reaching maximum height (T’2, min). The parameters obtained from the gas release curve were the maximum height of gaseous release (H’m, mm), the time to reach maximum gas release (T’1, min), the total quantity of gas produced (VTOT, mL), retained (VRET mL) and released (VREL, mL) in the dough at the end of the test, and finally, the gas retention coefficient (RC), calculated as VRET/VTOT (%). Analyses were performed in duplicate.

### 2.8. Bread Measurements

#### 2.8.1. Moisture Content

The Official Standard Method AACC 44–15.02 [39] was followed for the determination of bread loaves’ moisture content. For each formulation, three repetitions per batch underwent the analyses.

#### 2.8.2. Specific Volume

The breads’ specific volume was calculated according to the AACC 10–05.01 [40] rapeseeds displacement method as the ratio of bread volume (mL) to bread weight (g). For each formulation, three repetitions per batch underwent the analyses.

#### 2.8.3. Textural Properties

A texture profile analysis (TPA) was used to determine the textural properties of the bread samples. The analysis was conducted using a texture analyzer (TA-XT plus, Stable Micro Systems, Surrey, UK) equipped with a 36 mm diameter cylindrical probe. Three bread slices from three breads per batch underwent a double-compression test with 50% depth and a constant speed of 1 mm/s and 30 s gap. The attributes considered were hardness, cohesiveness, springiness, resilience, and chewiness, which were obtained from the registered curves.

#### 2.8.4. Color Determination

Crust and crumb color were evaluated using a tristimulus colorimeter (Minolta CR-300, Konica Minolta Sensing, Osaka, Japan) with a CR-300 measuring head on the day of baking. Crust color was evaluated from the distal and central parts of three loaves for each sample, while crumb color was measured at the center point of two middle slices of each of the three loaves. The instrument was calibrated prior to evaluation. The results were expressed based on the CIELab measuring system and the following parameters were obtained: L* (black when L* = 0 or white if L* = 100), a* (−a* means greenness and +a* redness), and b* (−b* means blue; +b* means yellow).

#### 2.8.5. Crumb Grain Characteristics

A digital image analysis system was used to evaluate crumb grain characteristics. Initially, 2D high-resolution images were acquired using an Epson Perfection V500 photo scanner (Epson, Suwa, Japan). Subsequently, analyses were conducted in duplicate on a 20 × 20 mm sub-images, which were taken from the center of the original images and then segmented via thresholding (using the Huang algorithm) to accurately segregate the gas and solid phases and define the distribution of cell and cell wall sizes. The data were obtained using Image J program (NIH, Rockville, MD, USA). The number of cells, cell to total area ratio, wall to total area ratio, and cells/cm^2^ were the essential features defining crumb grain [41]. In order to classify cell area distribution and cell number distribution, the following previously selected four-dimensional categories were adopted: Class 1 (<1.0 mm^2^); Class 2 (0.1–0.99 mm^2^); Class 3 (1.0–9.9 mm^2^); Class 4 (10–40 mm^2^).

### 2.9. Determination of Soluble, Insoluble, and Total Polyphenol Fractions

Soluble (SP) and insoluble (IP) phenolic compounds were assessed in both bran and bread samples following the procedures previously described by Conte et al. [42], with slight modifications. Briefly, for the determination of the soluble fraction, 4 mL of 37% hydrochloric acid–methanol –water (1:80:10, *v*/*v*/*v*, Carlo Erba Reagents, Ltd., Milan, Italy) was added to a 1 g aliquot of both bran and partially dried and ground breads. The samples were vigorously shaken for two hours at room temperature and then centrifuged (2500× *g*, 3 min). The supernatant was collected, and the remaining residue underwent a second extraction following the same procedure. Supernatants from both extractions were filtered through cellulose acetate syringe filters with a pore size of 0.45 μm (LLG Syringe Filter CA, Carlo Erba, Milano, Italy) and combined for SP determination. To measure the insoluble polyphenol fraction, the residual material was mixed with 5 mL of a methanol–concentrated sulfuric acid (10:1, *v*/*v*, Carlo Erba Reagents, Ltd., Milan, Italy) solution, incubated in a shaking water bath (85 °C for 20 h), and centrifuged at 3500× *g* for 10 min. Then, supernatants were filtered through cellulose acetate syringe filters with a pore size of 0.45 μm (LLG Syringe Filter CA, Carlo Erba, Milano, Italy) and stored at room temperature before readings. Extractions were performed in triplicate and the extracts obtained were analyzed using the Folin Ciocalteu (Carlo Erba Reagents, Ltd., Milan, Italy) protocol in a spectrophotometer (Spectrophotometer, Hewlett–Packard, PaloAlto, San Francisco, CA, USA) set at 750 nm. Total polyphenols concentration was obtained by summing the SP and IP fractions. Calibration curves were made using gallic acid as standard and the results were expressed as mg of gallic acid equivalent (GAE) per 100 g of bread (or bran) dry matter (dm). All analyses were performed in duplicate.

### 2.10. Determination of Antioxidant Activity

The antioxidant capacity of the experimental samples of bran and bread samples was determined using the ABTS·^+^ spectrophotometric assay. This is based on the ability of antioxidant compounds to reduce the radical cation ABTS^+^ to ABTS, which depends on their concentration, antioxidant power, and time of reaction [43]. Briefly, a 2 g aliquot of bran (3 g of bread samples) was shaken at room temperature for 1 h with 20 mL (10 mL for bread) of a methanol–water solution (50:50, *v*/*v*, Carlo Erba Reagents, Ltd., Milan, Italy) acidified (to pH 2) with 1 M hydrochloric acid (Carlo Erba Reagents, Ltd., Milan, Italy). The obtained samples were centrifuged at 2500× *g* for 10 min and the supernatant was recovered. Then, the residues were extracted again following the same procedure but using 20 mL (10 mL for bread) of an acetone–water (70:30, *v*/*v*, Carlo Erba Reagents, Ltd., Milan, Italy) solution. At this point, the methanolic and acetonic extracts were combined, made up to 25 mL with methanol, and used for the determination of the ABTS radical-scavenging activity. To this end, ABTS was dissolved in phosphate buffer to produce a stock solution with a final concentration of 7.4 mM. The radical cation was prepared by reacting equal volumes of the 7.4 mM ABTS stock solution and 2.6 mM potassium persulfate solution in the dark at room temperature for 12 h. Immediately before use, ABTS·^+^ was diluted with phosphate buffer to obtain a working solution with an absorbance of 1.0 ± 0.2 at 734 nm. Subsequently, a 40.8 μL aliquot of each experimental sample was made to react with 2 mL of the freshly prepared ABTS^+^ working solution and the absorbance values were recorded after 6 min at 734 nm (22 °C). A Trolox standard curve was used to quantify the ABTS radical scavenging activity, and the results were expressed as μmol of Trolox (Sigma-Aldrich, Milan, Italy) equivalent (TE) per 1 g of sample (dm). All measurements were taken in triplicate.

### 2.11. Sensory Evaluation

An attribute diagnostics test was conducted to determine consumer responses to specific attributes in the bran-fortified breads, utilizing hedonic ratings as a quantitative affective measure. This type of test involves asking judges questions about the intensity of certain attributes, which can help to understand the reasons for any preference or rejection of the product [44]. This sensory evaluation considered bread samples enriched with five of the seven wheat bran varieties under study: the improved emmer variety GP, the improved einkorn variety NOR, the landrace GAR, the old wheat CAP and the ancient wheat KHO. Bread samples from PP and MON were excluded as GP and NOR showed better technological behavior. A panel of 73 participants, including students and staff of the Department of Agricultural Sciences at Sassari University (Italy), aged between 18 and 60, were enlisted to rate the five composite breads. The participants were asked to rate specific attributes of the breads, such as crumb color, odor, and consistency, on a scale from 1 to 9 (1 = less intense; 9 = more intense). They were also asked to express their overall liking of the breads on a 9-point hedonic scale (1 = extremely dislike; 9 = extremely like). The test was conducted in a laboratory [45] using individual booths, in accordance with ISO 8589 standards [46]. Data were collected using Smart Sensory Box software (version 2.11.6, Smart Sensory Solutions, S.r.l., Sassari, Italy). Functional breads were prepared the day before the sensory analysis. Afterwords, they were sliced, and each slice was divided into four equal pieces. These pieces were then presented to the participants in a randomized and balanced order.

### 2.12. Statistical Analysis

Statistica 12.0 software (StatSoft, Inc., Tulsa, OK, USA) was used to perform all the statistical tests. One-way analysis of variance (ANOVA) with Tukey’s honestly significant differences (HSD) post hoc test (*p* < 0.05) was adopted to investigate significant differences between each pair of means. For the sensory evaluation, the differences among bread samples were identified using main effect ANOVA, followed by an HSD post hoc test (*p* < 0.05). Pearson correlation analysis was also employed to examine the relationships between the selected parameters.

## 3. Results and Discussion

### 3.1. Proximate Chemical Composition of Wheat Brans

Bran samples from different wheat species showed significant differences (*p* < 0.05) in terms of their chemical composition (Table 2). The bran of the improved einkorn genotypes MON and NOR were clearly distinct from all the other genotypes, with the highest ash, fat, and TDF contents (Table 2). Notably, significant variability was observed within brans derived from genotypes classified under the same species, GP and PP. The first sample had higher protein content, while the second sample had higher levels of ash, fat, and TDF. GAR, CAP, and KHO brans had significantly (*p* < 0.05) lower protein and ash contents. Nonetheless, the former exhibited significantly (*p* < 0.05) higher fat content and lower TDF content, whereas CAP and KHO brans showed the opposite trend (Table 2). It is noteworthy to mention that, despite having different macronutrient contents, brans from all studied wheat species contained more protein, ash, and lipid compared to common wheat bran [30,47]. Accordingly, these grains have the potential to fortify bakery products and enhance their nutritional profile. No studies were found in the literature concerning the chemical composition of any ancient grain bran. However, Mefleh et al. [48] studied the chemical composition of the flour from the same set, and found similar results, except for the ash content.

### 3.2. Particle Size Distribution

**Figure 1:**
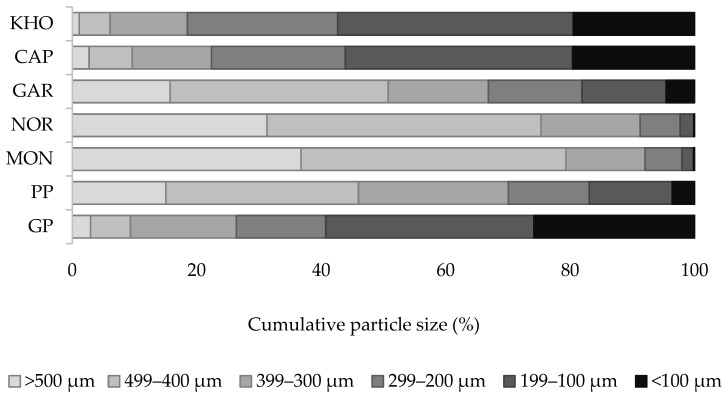
Cumulative particle size distribution of the studied wheat bran genotypes.

Figure 1 illustrates the cumulative particle size distribution for each studied bran. The results evidenced that, despite the reduction in the bran samples when using the same milling method, their particle size did not result in uniform compositions. Specifically, the wheat bran genotypes that were studied were classified into two categories. The first category, which included the improved einkorn varieties MON and NOR, was composed mainly of particles larger than 300 µm with percentages of 92.04% and 91.24%, respectively. PP and GAR samples followed, with 70.07% and 66.85%, respectively. The second category, which includes KHO, CAP, and GP genotypes, had bran mainly composed of fractions smaller than 300 µm, with percentages of 81.52%, 77.63%, and 73.63%, respectively. These observed differences were likely due to the inherent grain characteristics, including grain hardness and bran composition [49]. It is important to highlight that a superfine particle composition can result in an increased surface area, which can influence physical or chemical interactions and consequently affect the quality of dough and bread [50]. This variability may have an impact on dough rheology, especially in relation to water absorption and, subsequently, on bread quality.

### 3.3. Water Retention Capacity

**Figure 2:**
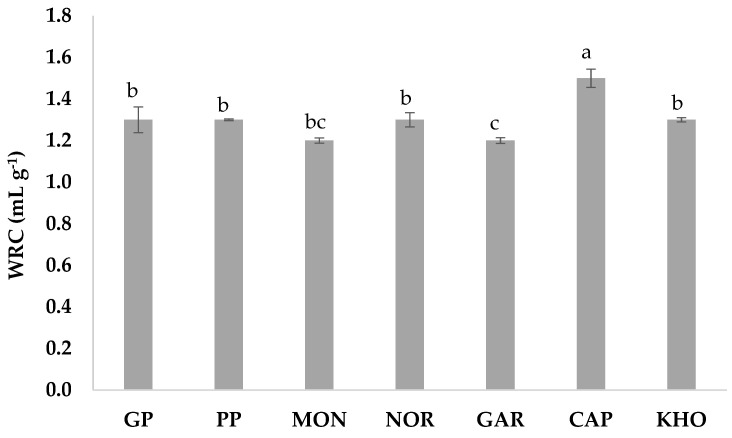
Water retention capacity (expressed in mL g^−1^) of the studied wheat bran genotypes. Histograms with the same letter do not differ significantly from each other according to Tukey HSD test (*p* < 0.05). Abbreviations: GP (Giovanni Paolo); PP (Padre Pio); MON (Monlis); NOR (Norberto); GAR (Garfagnana); CAP (Cappelli); and KHO (Khorasan).

Figure 2 presents the hydration property properties of the different ancient grain brans, as determined by the amount of water they are able to retain in the presence of an external force. The values obtained indicated that although the observed differences among the WRCs of the different wheat brans were minor, they were nevertheless significant (*p* < 0.05) for two samples, namely CAP bran and GAR bran, which exhibited the highest (1.45 mL g^−1^) and lowest ability (1.17 mL g^−1^) to retain water, respectively. These findings may contradict previous research suggesting that a larger particle size correlates with higher water retention capacity in wheat bran [51]. However, in this study, the contradiction does not seem to be linked to particle size distribution. Specifically, the other studied wheat bran genotypes categorized under the same particle size distribution classes as CAP and GAR brans (fine and large particle size distributions, respectively) did not exhibit significant differences in terms of water retention capacity (WRC). It is also worth noting that the range of particle size distribution used in this study differs from that reported in other studies [51,52]. Clearly, particle size distribution is not the sole crucial factor influencing bran WRC. Other factors, such as bran’s chemical composition, particularly soluble and insoluble fibers, along with the physical and structural changes resulting from the sample preparation methods, could also contribute to the observed results.

### 3.4. Dough Measurements

#### 3.4.1. Dough Mixing Properties

The studied doughs showed significant differences (*p* < 0.05) in all mixing parameters (Table 3). The enrichment of common wheat dough with the studied ancient wheat brans resulted in a significantly higher (*p* < 0.05) WA compared to the control. This finding aligns with the results from several studies, which attributed the increased WA to the dietary fiber in bran [50,53,54]. The higher number of hydroxyl groups in dietary fiber in comparison to other components of wheat flour (starch and protein) facilitates its capacity to bind a greater amount of water through hydrogen bonding, thereby enhancing WA [53]. The NOR, MON, and GAR bran doughs required the longest time to reach maximum dough consistency, exhibiting the highest DDT values among the enriched doughs. These three bran types primarily consist of large particles (Figure 1), which may account for the extended DDT. This observation is consistent with the findings reported by Zhang et al. [20], who observed that the incorporation of coarse wheat bran resulted in prolonged mixing times compared to finer bran. However, it is noteworthy that only the NOR bran dough showed a significant difference (*p* < 0.05) in DDT compared to the control dough. This outcome could be attributed to the significantly higher (*p* < 0.05) TDF content of this bran compared to all other samples (Table 2), which may impair the gluten network structure, which ultimately requires more time to reach maximum consistency. The highest stability value, coupled with the lowest fall in consistency, was observed in GAR bran dough, with a stability time of 20 min and a dough-softening value of 5.5 BU. The addition of emmer and einkorn wheat bran to dough formulations resulted in ST times ranging from 19.1 to 20 min. However, no significant differences (*p* < 0.05) were found between these samples and the control, either for ST or DS (Table 3). In contrast, CAP and KHO bran doughs exhibited significantly lower stability times (12.2 min and 11 min, respectively) compared to both the control and the other composite samples (*p* < 0.05). Additionally, dough enriched with KHO showed the highest DS value among all samples, which was only significantly different (*p* < 0.05) from GAR bran dough. This observation aligns with the findings reported in several studies that demonstrated an increase in dough ST with an increase in bran addition [53,54,55]. In contrast, other authors noted higher DS and lower dough ST values, attributing this to the negative impact of fiber–protein interactions, which form a weaker and less stable gluten network [50,56,57]. Similarly, Lin et al. [58] observed a decrease in dough ST with the addition of 20% fine bran particles. Therefore, the combination of a high TDF content and the fine particle size distribution of CAP and KHO bran may explain their significantly lower (*p* < 0.05) ST values.

#### 3.4.2. Dough Viscoelastic Properties and Extensibility Measurements

According to Table 4, significant differences (*p* < 0.05) were observed among the studied wheat bran genotypes in all dough viscoelastic parameters. The storage modulus (G′) was greater than the loss modulus (G″) in all the enriched dough samples and the control sample, which indicates their predominant elastic behavior, meaning their ability to store and recover energy upon deformation. However, no significant differences (*p* < 0.05) were observed in the constant tan δ (G″/G′) in all samples, suggesting an equal contribution of the elastic and viscous components to the rheological properties of the doughs with the incorporation of bran. Among the enriched samples, doughs composed of GP and KHO bran showed the highest moduli values, followed by GAR, PP, and CAP bran doughs, and finally, doughs containing bran from the improved einkorn varieties MON and NOR. However, both moduli values of the composite doughs were significantly (*p* < 0.05) higher than those of the control dough. These findings suggest that the addition of bran to dough samples exhibited enhanced viscoelastic characteristics, likely due to an appropriate interaction between gluten proteins and bran fibers, which contributed to a more stable network structure. This can be ascribed to two possible factors: firstly, to the presence of fibers within the viscoelastic matrix, functioning as fillers or alternatively, to the reduction in lubrication provided by water due to the competition between gluten and fiber for this crucial component [53,59]. Other studies explained that, upon water absorption, both gluten proteins and bran fibers expanded to each form a continuous film-like matrix. Eventually, the two matrixes entangled and cross-linked to create a highly elastic gluten–fiber structure [60].

Extensibility measurements demonstrated that all composite doughs, except those produced with MON and NOR brans, exhibited significantly (*p* < 0.05) higher resistance to extension (R) values compared to the control (Table 4). The addition of ancient wheat brans led to a significantly (*p* < 0.05) decreased extensibility (E) compared to the control (Table 4). Similar findings were reported in several studies attributing the reduced E to the negative impact of bran fibers on the gluten network, which disrupts the starch–gluten matrix [54,61,62]. Among the experimental samples, dough enriched with GP bran showed the highest ability to stretch without breaking, proceeded by GAR and KHO bran doughs, and then the remaining varieties.

Although dough elasticity and extensibility derive from two distinct methods of analysis, and they are not usually linked, the dough enriched with the ban of GP emmer wheat showed the most elastic behavior, with the highest ability to extend. This elasticity–extensibility balance characterizing the GP bran dough could be the result of a combination of factors, such as its TDF content and particle size distribution. In effect, the low content of the former (Table 2) can lead to a minor fiber–gluten interaction, which confers to a higher elasticity. This is consistent with previous reports that noted the negative effect of fibers on gluten network [49,50]. Moreover, the fine particle sizes in this modern emmer wheat (Figure 1) could be a significant factor enhancing its dough extensibility and resistance to extension. Several authors reported that both R and E increased with the reduction in whole wheat flour particle sizes, whether in the case of doughs prepared for bread-making [49], for crackers [63], or for tortillas [64].

#### 3.4.3. Rheofermentometer Analysis

Functional doughs exhibited significant differences (*p* < 0.05) in their fermenting properties (Table 5). With regard to the parameters associated with dough development, all bran-enriched doughs had a significantly (*p* < 0.05) lower maximum dough height and shorter T’2 than the control dough. Within the experimental samples, doughs enriched with GP and NOR brans demonstrated the highest Hm, reaching 26.05 mm and 25.75 mm, respectively. These were followed by doughs enriched with CAP, KHO, MON, and GAR brans, while PP bran dough showed the lowest height (19.1 mm). Gomez et al. [54] reported a similar decreasing effect of extruded wheat bran on Hm. This effect could primarily be attributed to the bran fibers, since their inclusion in dough formulations reduced the dough’s maximum height, as also noted by Wang et al. [65]. The CAP bran dough presented the highest structural stability, as indicated by its significantly (*p* < 0.05) longer T’2 of 52 min, compared to the other functional doughs. The times at which these latter began to collapse (T’2) ranged from 38.15 min, observed in both GP and NOR bran doughs, to 29 min, recorded for PP bran dough. Although there was discrepancy in the literature regarding the influence of wheat bran particle size on dough and bread quality, several studies demonstrated that incorporating finely milled bran into wheat dough formulations enhanced gluten development and strengthened the dough [58,66]. This trend was evident in the functional doughs enriched with brans with the finest particle size, such as GP, CAP, and KHO, which exhibited the highest Hm values and the longest T’2. However, in the case of NOR bran dough, the high values of these parameters were probably due to its high protein content rather than its particle size.

With regard to the gas release parameters, the studied doughs exhibited a maximum gas release height (H’m) ranging from 80.75 mm to 64.90 mm, which was not significantly (*p* < 0.05) different from the dough control. Doughs enriched with MON, NOR, KHO, and GP brans demonstrated significantly higher H’m values (*p* < 0.05) in comparison to the other samples, while the lowest value was observed in dough enriched with CAP bran. Only CAP, NOR, and PP dough brans obtained a significantly (*p* < 0.05) higher (T’1) than the control dough. In fact, the CAP bran dough showed the slowest fermentation process, as evidenced by the significantly longer time it required to reach the maximum height of gaseous release (T’1) (*p* < 0.05), at 129.45 min. Similarly, doughs prepared with NOR and PP bran showed extended T’1 values, recorded at 120 and 114.45 min, respectively. In contrast, doughs prepared with MON and GAR bran along with the control presented moderate fermentation times, with T’1 values of 99.45 min, 81.45 min, and 85 min, respectively. The fastest fermentation times were observed in doughs enriched with GP and KHO brans, which achieved T’1 values of 62.15 min and 54.45 min, respectively. With regard to the production of gas, all bran-enriched doughs demonstrated a significantly (*p* < 0.05) higher total quantity of gas produced than the control, except for PP and KHO bran doughs. The GP bran dough exhibited the highest value for the total quantity of gas produced (1839 mL), which was significantly higher (*p* < 0.05) than that observed in all other samples. This was followed by the NOR bran dough, (1722 mL), and then the remaining doughs, which demonstrated a significantly (*p* < 0.05) lower gas production, with the lowest values recorded for the PP (1580 mL) and CAP (1512 mL) bran doughs. These findings corroborate those of the dough development results, as the doughs showing the best dough development also demonstrated the highest yeast activity and gas production, with the exception of CAP. This discrepancy could be attributed to the possible physical interactions between the CAP bran and the gluten network reducing its ability to retain gas effectively during fermentation. Functional doughs with the highest gas production, namely GP and NOR bran doughs, also released significantly larger quantities of gas (VREL, *p* < 0.05), whereas PP and CAP bran doughs, which had the lowest gas production, released significantly smaller amounts (*p* < 0.05). Besides PP bran dough, all the studied doughs released a significantly (*p* < 0.05) higher amount of gas than the control. While these results indicate a superior gas retention capacity in PP and CAP bran doughs, as further supported by their significantly (*p* < 0.05) higher retention coefficients (RC) of 78.85% and 79.60%, respectively, it is important to note their inherently low level of gas production. The observed differences in the fermenting properties of the bran-enriched doughs can be attributed to a combination of factors intrinsically linked to the bran characteristics, including their particle size distribution and the composition and type of fibers present. For instance, Wang et al. [65] found that the physical disruption of the gluten protein matrix, induced by the inclusion of different types of fibers, resulted in an increase in gas release. This is consistent with our results, where MON, NOR, and KHO bran doughs had a significantly (*p* < 0.05) higher TDF content and increased gas release compared to the other samples. Conversely, in the case of GP bran dough, the primary factor could be that the highest levels of carbon dioxide production were obtained, which may have counteracted its superior performance in terms of viscoelasticity and extensibility. This would result in a reduction in tolerance to physical deformation during the fermentation process, and consequently, an unexpectedly low RC.

### 3.5. Bread Analyses

#### 3.5.1. Chemical Analysis

Statistical analysis showed significant differences (*p* < 0.05) between the fortified and the control bread in terms of chemical composition (Table 6). As expected, a noteworthy improvement in bread’s macronutrients content was detected in different amounts depending on the bran genotype. In effect, bread partially composed with the improved einkorn varieties MON and NOR exhibited the greatest protein, ash, and fat contents, followed by bread fortified with improved emmer varieties GP and PP. For TDF, bread fortified with ancient wheat KHO obtained the highest contents. Some variations were noticed between the bran’s chemical composition and their respective breads. For example, MON bran showed a high TDF content, whereas its bread had a lower TDF content than expected. On the contrary, GP bran, which exhibited the lowest TDF content, obtained a higher TDF content in the bread. This could be attributed to the interactions of certain components during the baking process.

#### 3.5.2. Bread Measurements

**Figure 3:**
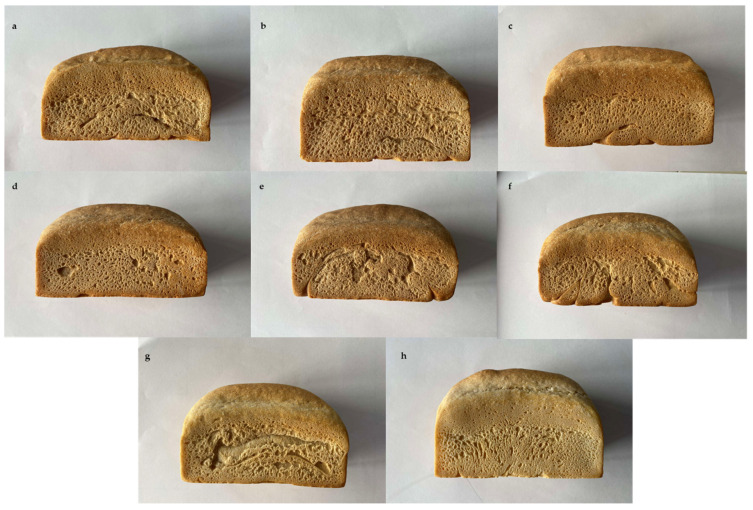
Images of the studied bran-enriched bread samples. (**a**): Giovanni Paolo; (**b**): Padre Pio; (**c**): Monlis; (**d**): Norberto; (**e**): Garfagnana; (**f**): Cappelli; (**g**): Khorasan; and (**h**): Control.

The specific volume (SV) and textural parameters of the bread provide a comprehensive understanding of the overall bread quality (Figure 3). Statistical analysis showed that all the bran-enriched breads had a significantly (*p* < 0.05) lower SV (from 2.46 ± 0.04 to 2.64 ± 0.01 mL g^−1^) than the control bread (2.97 ± 0.03 mL g^−1^) (Table 7). Such results were expected considering the great amount of TDF that was initially present in the genotypes’ brans (Table 2), which can potentially disrupt the gluten network, leading to reduced loaf expansion and a subsequent reduction in SV. This negative effect was also observed by Almeida et al. [31]. Among the composite breads, the lowest SV was observed in GP–bran bread. Such results can be potentially related to its high surface area due to its fine particle size distribution (Figure 1), as previously reported by Noort et al. [50]. Their findings confirmed the deleterious impact of the increased surface area on bread volume reduction.

Textural properties refer to the physical characteristics that affect the mouthfeel and the bread structure. Typically, breads that are characterized by a soft and tender texture have more appealing qualities. It is well known in the literature that the partial substitution of wheat flour with bran results in physical and chemical effects on the gluten structure, which are strongly correlated with bread softness [67,68]. Accordingly, there were statistically significant differences (*p* < 0.05) between the functional breads and the control bread in terms of hardness and chewiness (Table 7). Breads produced from improved emmer genotypes of brans GP and PP exhibited significantly higher hardness values (17.00 ± 0.75 and 18.98 ± 1.33 N, respectively) compared to the control bread (11.44 ± 0.07 N). With regard to chewiness, the bread produced with PP bran (15.39 ± 1.20) exhibited a statistically significant difference (*p* < 0.05) from the control bread (9.99 ± 0.16). In practical terms, bread with a harder structure requires a greater expenditure of effort to be chewed. This was corroborated by the findings of the present study in the case of bread made with PP bran, but not with that made with GP bran. This discrepancy is likely attributable to a combination of factors that are exclusive to the former case. Indeed, a comparison of the GP and PP bran breads revealed that the second had a larger particle size distribution (Figure 1), which can hinder the formation of the gluten network, resulting in a denser crumb and consequently increasing hardness and chewiness. Similar trends were observed by Lin et al. [49]. Furthermore, the dough formed with the PP genotype bran exhibited the lowest extensibility (Table 4), confirming its lack of elasticity, which can subsequently result in the production of harder and chewier bread loaves. The findings of the present study corroborate those of Sun et al. [69], who found a correlation between low extensibility and elevated hardness and chewiness.

Color parameters are other crucial factors that interfere with bread acceptability. The fortification of bread with different brans resulted in a notable alteration in its crust and crumb color indices (*p* < 0.05) (Table 8). All the functional breads presented a darker (lower lightness L*) and more reddish crust in comparison to the control bread (a* positive). A similar trend was observed for the crumb color indices, where all the bran-fortified breads had a lower lightness (L*) and a higher tendency toward redness (a* positive) and yellowness (b* positive) in comparison with the control bread (Figure 3). The Maillard and caramelization reactions are the main inducers of the browning of the crust during the baking process. They are enhanced by the presence of amino acids and reducing sugars, which are profusely available in wheat bran [67], which can explain the darker aspect of the bran-enriched breads. This darkening effect was reported in previous studies [70,71]. Heiniö et al. [72] primarily attributed this to the high ash content in bran and its content of yellow pigments. Among the bran-enriched breads, the one made of NOR bran showed a significant difference (*p* < 0.05) in the darkness of its crust and crumb, presenting the lowest lightness L* values (Table 8).

**Figure 4:**
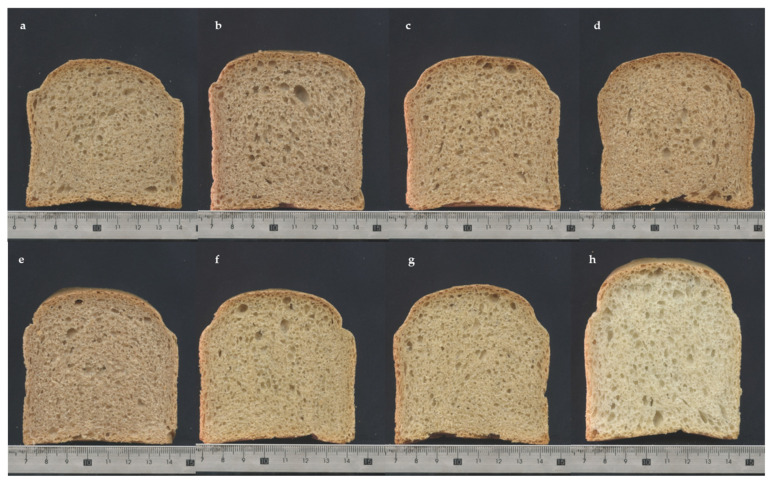
Images of the crumb of the studied bran-enriched bread samples. (**a**): Giovanni Paolo; (**b**): Padre Pio; (**c**): Monlis; (**d**): Norberto; (**e**): Garfagnana; (**f**): Cappelli; (**g**): Khorasan; and (**h**): Control.

**Figure 5:**
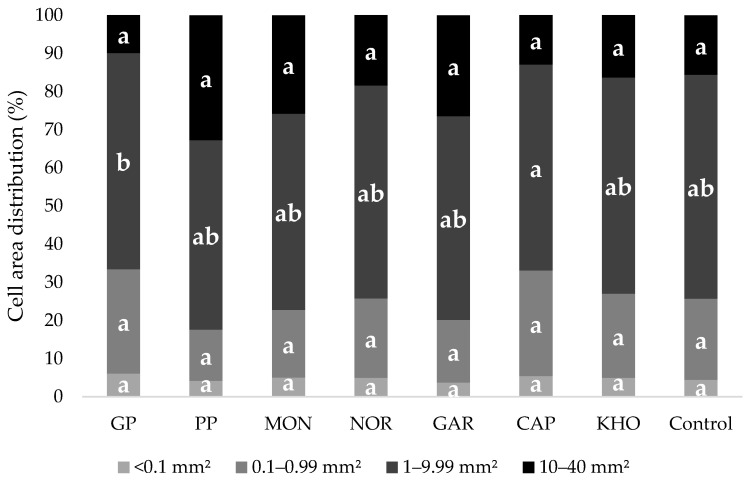
Cell area distribution of control and fortified breads as percentage of the total area according to the four pre-selected dimensional categories: Class 1 (<1.0 mm^2^); Class 2 (0.1–0.99 mm^2^); Class 3 (1.0–9.99 mm^2^); Class 4 (10–40 mm^2^). Histograms with the same letter do not differ significantly from each other according to Tukey HSD test (*p* < 0.05). Abbreviations: GP (Giovanni Paolo); PP (Padre Pio); MON (Monlis); NOR (Norberto); GAR (Garfagnana); CAP (Cappelli); and KHO (Khorasan).

Another important factor influencing the final quality of the bread is the grain structure of the crumb (Figure 4). The present study did not identify any statistically significant differences between the bran-fortified breads and the control bread in the following cell area distribution classes: class 1 (<1.0 mm^2^), class 2 (0.1–0.99 mm^2^) and class 4 (10–40 mm^2^). However, in the third class (1.0–9.9 mm^2^) only GP and CAP bran breads were significantly (*p* < 0.05) different from each other. Therefore, the fortification of common wheat bread with ancient grain brans did not result in a notable impact on the crumb pore uniformity and cell distribution (Figure 5).

### 3.6. Polyphenol Fractions and Antioxidant Activity of Bran and Bread Samples

Phenolic compounds are plant secondary metabolites that exhibit noteworthy antioxidant properties that may potentially contribute to various health benefits. It has been demonstrated that approximately 83% of the total phenolic content (TPC) of wheat is concentrated in the outer layers of the kernel, predominantly comprising the bran fraction [45].

Table 9 and Table 10 summarize the results of soluble, insoluble, and total polyphenol fractions of the seven studied brans and the resulting fortified breads. All the ancient grain brans displayed a notable amount of TPC, ranging between 517 ± 9 and 382 ± 7 mg GAE 100 g^−1^ dm. These concentrations were found to be higher than those previously reported by other authors [73,74,75] for various wheat bran cultivars, thereby confirming that the by-products of ancient grains could be a natural and rich source of phenolic compounds. Among the studied bran material, bran derived from the improved einkorn genotypes NOR and MON contained the highest total amount of polyphenols, which accounted for 517 ± 9 and 506 ± 3 mg GAE 100 g^−1^ dm, respectively. The improved emmer genotypes PP and GP bran were subsequently ranked, followed by the remaining genotypes, with the KHO bran exhibiting the lowest amount of total polyphenols (382 ± 7 mg GAE 100 g^−1^ dm). As expected, the insoluble bound phenolic content exceeded that of the soluble free extractable phenolic fraction in all bran genotypes (Table 9). This trend has also been confirmed in other studies on modern wheat bran [73,76]. However, it is worth mentioning that the bran of the improved einkorn genotypes MON and NOR had the lowest average ratio of insoluble to soluble polyphenols (IP/SP), indicating a better polyphenol composition compared to the bran of the other genotypes. This underlines their superiority, not only in terms of TPC amount, but also in terms of quality. The chief advantage is that the free polyphenol fraction is more readily bioaccessible for absorption, while the bound fraction is characterized by a limited bioaccessibility, restricting its potential bioactivities [77,78]. Table 9 shows that the incorporation of the selected bran genotypes had a significant impact (*p* < 0.05) on the polyphenol fractions of the resulting breads. This is evidenced by the higher TPC values observed in all the fortified samples, which ranged from 342 ± 5 mg GAE 100 g^−1^ dm in the KHO-fortified bread to 398 ± 1 mg GAE 100 g^−1^ dm in the MON-fortified bread, compared to the control (281 ± 5 mg GAE 100 g^−1^ dm). This finding is consistent with the previously observed trend in the bran samples and was further confirmed in terms of polyphenolic composition. In fact, the partial substitution of the common wheat flour with the studied brans significantly affected both the soluble and insoluble polyphenols of all fortified breads, with a more pronounced increase in the extractable fraction. A comparison of the samples with the control revealed that the breads prepared with the improved einkorn genotype MON exhibited the most significant increase. In particular, the soluble and insoluble fractions rose by 136% and 31%, respectively. The NOR bread and PP bread also exhibited a significant increase in both soluble and insoluble polyphenols, with a rise of 120% and 30% and 115% and 26%, respectively. In contrast, the GP, GAR, CAP, and KHO breads demonstrated only slight improvements, with the latter bread exhibiting the least significant increases in both fractions (+67% for the soluble and +17% for the insoluble). A comparison of the present results with those of previous studies is challenging, given the lack of data on the phenolic composition of ancient wheat bran and bread products. Nonetheless, the adopted fortification method has proven effective in increasing the readily available soluble fraction in wheat bread.

Phenolic compounds represent the primary natural reservoir of antioxidants in cereal grains. They have been reported to offer major nutritional and therapeutic benefits, encompassing anti-allergic, antiviral, anti-inflammatory, and anti-mutagenic properties [79]. The antioxidant capacity of each sample was evaluated by measuring its ability to scavenge the ABTS radical cation relative to the standard (Trolox). As illustrated in Table 9, significant differences (*p* < 0.05) in antiradical activity were observed among the studied bran samples. The improved einkorn genotypes, NOR and MON brans, exhibited the highest antiradical activity (about 107 and 105 μmol TE g^−1^ dm, respectively), followed by the improved emmer GP and PP brans. The remaining genotypes exhibited reduced activity, with the KHO bran recording the lowest value (about 46 μmol TE g^−1^ dm). This trend closely mirrors that observed in the TPC, confirming the direct relationship between antiradical activity and phenolic fractions, as reported in other studies [80,81]. Indeed, Pearson correlations were used to explore these consistent findings, revealing high correlation coefficient values (r). A highly positive correlation was found between higher levels of antiradical activity and increased soluble (r = 0.91; *p* < 0.0001), insoluble (r = 0.82; *p* < 0.0001), and total (r = 0.96; *p* < 0.0001) polyphenol contents. In the case of the fortified breads, all composite samples displayed an enhanced antioxidant capacity, with statistically significant values (*p* < 0.05) compared to the control bread (Table 10). The breads enriched with NOR and PP brans demonstrated the highest and lowest measured antioxidant activities, respectively, with values of 72 μmol TE g^−1^ dm and 48 μmol TE g^−1^ dm. It is noteworthy that, despite their initially high polyphenol content, both the MON bran and PP bran breads ranked at the bottom of the list, alongside the KHO bran bread. Given that phenolic and antioxidant content are thermolabile, it is plausible that excessive temperatures during the baking process may have diminished the free radical scavenging action of the phenolic compounds present in these two functional breads, or may have led to the degradation of the already-formed antioxidants. Nevertheless, further research is necessary to substantiate this hypothesis. While the antioxidant content of the other fortified breads was in line with their respective phenolic content (Table 10), Pearson correlations only revealed a significant positive correlation with relatively low coefficient values for the insoluble and total polyphenols (r = 0.49; *p* < 0.05 for insoluble and r = 0.45; *p* < 0.05 for total polyphenols).

### 3.7. Sensory Evaluation

**Figure 6:**
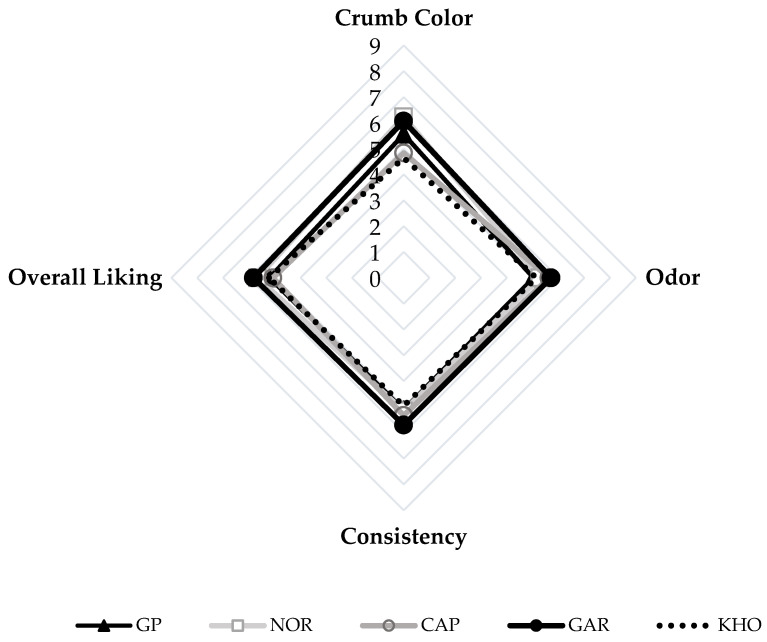
Spider graphs of the sensory attributes and overall liking of the five selected fortified breads.

The statistical analysis, employing a main effect ANOVA, of the sensory evaluation data shows significant differences (*p* < 0.05) among the tested samples with regard to the attributes of crumb color, odor, consistency, and overall liking (Figure 6).

**Figure 7:**
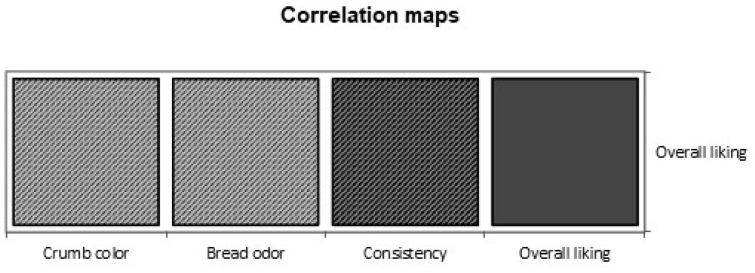
Correlation maps of the sensory attributes (the more intense the color, the stronger the correlation).

The first attribute, evaluated using an intensity scale, was the color of the crumb. The darkest crumb color was identified by consumers as being present in bread fortified with the GAR and NOR brans, while breads fortified with CAP and KHO bran were identified as having the lightest crumb color. The GP bran bread was significantly different (*p* < 0.05) from both groups and was classified in the middle. With regard to the odor attribute, only GAR and GP bran breads were significantly (*p* < 0.05) different from each other. In fact, participants rated the former as having the most intense aroma and the latter as having the least intense aroma. In terms of consistency, bread made with GAR bran was perceived as the most consistent compared to the other bread samples, which were not significantly different from each other. For the overall liking, the GAR and NOR bran breads received the highest scores among the fortified breads. However, they were only significantly (*p* < 0.05) different from KHO bran bread, which was the least preferred. Both GP and CAP bran breads were positioned in the middle and did not demonstrate statistical differences from the aforementioned samples. It is worth highlighting the favorable sensory profile of the emmer bran breads GAR and NOR, as evidenced by their superiority in all attributes. To assess the major contributing attributes to this significant preference, Pearson correlations were performed. The findings revealed positive correlations between all diagnostic attributes and the overall liking, with consistency exhibiting the highest correlation (Figure 7).

## 4. Conclusions

The renewed interest in ancient grains has been driven by several factors, including health and nutritional considerations, environmental concerns, consumer preferences, and awareness of the importance of preserving traditional agricultural practices. In this study, the optimization of common wheat dough and bread with a 20% content of bran of ancient grains’ genotypes generally enhanced their nutritional profile without necessarily altering their properties. Regarding the rheological properties of the dough, all composite doughs maintained a desirable DDT and ST. They also showed an improved elastic characteristic and a decreased extensibility compared to the control dough. Concerning bread measurement results, all the bran-enriched breads showed a lower SV and a darker crust and crumb when assessed to the control bread. However, not all demonstrated a harder and chewier loaf texture. An overall improvement was noticed in terms of bread’s chemical composition and nutritional profile, specifically an enrichment in TDF and polyphenols content.

This study suggests that although ancient wheats are primarily used as wholemeal flours, their brans can be effectively added to refined flour-based bread formulations to create functional products with enhanced nutritional benefits. Incorporating ancient wheat brans into bread offers an opportunity to diversify formulations while supporting traditional agricultural practices and promoting biodiversity. However, to fully assess the benefits of their application to dough and bread formulations, future research should include a direct comparison with modern wheat bran. Additionally, a further exploration of the broader applications of ancient wheat brans in other bakery products or food matrices could expand their potential role in modern diets, highlighting their versatility and nutritional advantages.

## Figures and Tables

**Table 1:** List of the studied wheat genotypes.

Wheat Type	Genotypes	Code	Scientific Names
Improved emmer	Padre Pio	PP	*T. turgidum* ssp. *dicoccum* Schubler var. Padre Pio
	Giovanni Paolo	GP	*T. turgidum* ssp. *dicoccum* Schubler var. Giovanni Paolo
Improved einkorn	Monlis	MON	*T. monocuccum* ssp. *monocuccum* var. Monlis
	Norberto	NOR	*T. monocuccum* ssp. *monocuccum* var. Norberto
Old wheat	Cappelli	CAP	*T. turgidum* ssp. *durum* (Desf.)
Emmer landrace	Garfagnana	GAR	*T. turgidum* ssp. *dicoccum*
Ancient wheat	Khorasan	KHO	*T. turgidum* ssp. *turanicum*

**Table 2:** Proximate chemical composition of the studied wheat bran genotypes (g 100 g^−1^ dm).

Samples *	Protein	Ash	Fat	TDF
GP	22.58 ± 0.56 ^a^	3.34 ± 0.12 ^d^	2.46 ± 0.31 ^c^	19.10 ± 0.20 ^f^
PP	19.78 ± 1.29 ^d^	4.85 ± 0.94 ^c^	4.01 ± 1.01 ^b^	22.89 ± 0.06 ^d^
MON	21.87 ± 0.07 ^b^	5.57 ± 0.16 ^b^	5.67 ± 0.38 ^a^	25.14± 0.35 ^b^
NOR	23.21 ±0.11 ^a^	6.28 ±0.18 ^a^	5.52 ±0.17 ^a^	27.17 ±0.05 ^a^
GAR	18.54 ± 0.12 ^e^	4.78 ± 0.03 ^c^	4.29 ±0.32 ^b^	21.97 ± 0.15 ^e^
CAP	20.40 ± 0.12 ^cd^	3.42 ± 0.05 ^d^	2.09 ± 0.11 ^c^	24.46 ± 0.34 ^bc^
KHO	18.56 ± 0.04 ^e^	3.42 ± 0.10 ^d^	2.28 ± 0.02 ^c^	24.27 ± 0.01 ^b^

* Mean values ± standard deviation, values with the same letter do not differ significantly from each other according to Tukey HSD test (*p* < 0.05). Abbreviations: GP (Giovanni Paolo); PP (Padre Pio); MON (Monlis); NOR (Norberto); GAR (Garfagnana); CAP (Cappelli); and KHO (Khorasan).

**Table 3:** Dough mixing parameters.

Samples *	Water Absorption (%)	Dough Development Time (min)	Stability (min)	Dough Softening (BU)
GP	59.8 ^a^	1.7 ± 1 ^b^	20.0 ± 0 ^a^	14.5 ± 2 ^ab^
PP	57.5 ^a^	1.4 ± 1 ^b^	19.2 ± 2 ^a^	19.0 ± 7 ^ab^
MON	57.5 ^a^	2.8 ± 2 ^ab^	19.1 ± 2 ^a^	8.0 ± 2 ^ab^
NOR	58.5 ^a^	5.1 ± 0 ^a^	19.1 ± 1 ^a^	11.0 ± 1 ^ab^
GAR	57 ^a^	2.1 ± 0 ^ab^	20.0 ± 0 ^a^	5.5 ± 0 ^b^
CAP	59 ^a^	1.4 ± 0 ^b^	12.2 ± 0 ^b^	16.5 ± 6 ^ab^
KHO	58.5 ^a^	1.4 ± 0 ^b^	11.0 ± 0 ^b^	22.5 ± 3 ^a^
Control	54 ^b^	1.8 ± 0 ^b^	19.0 ± 0 ^a^	9.0 ± 0 ^ab^

* Mean values ± standard deviation, values with the same letter do not differ significantly from each other according to Tukey’s HSD test (*p* < 0.05). Abbreviations: GP (Giovanni Paolo); PP (Padre Pio); MON (Monlis); NOR (Norberto); GAR (Garfagnana); CAP (Cappelli); and KHO (Khorasan).

**Table 4:** Doughs’ viscoelastic properties and extensibility parameters.

	Viscoelastic Parameters			Extensibility Parameters	
Samples *	G′ (Pa)	G″ (Pa)	Tan δ	R (N)	E (mm)
GP	26,388 ± 1268 ^a^	9658 ± 332 ^a^	0.367 ± 0.001 ^ab^	0.15 ± 0.02 ^a^	48.07 ± 0.04 ^b^
PP	22,267 ± 65 ^bc^	8057 ± 466 ^bcd^	0.362 ± 0.020 ^b^	0.17 ± 0.01 ^a^	34.51 ± 0.60 ^d^
MON	18,150 ± 32 ^de^	7319 ± 175 ^de^	0.405 ± 0.001 ^a^	0.10 ± 0.00 ^b^	35.77 ± 0.12 ^d^
NOR	17,101 ± 815 ^e^	6709 ± 114 ^e^	0.393 ± 0.001 ^ab^	0.11 ± 0.00 ^b^	33.78 ± 0.02 ^d^
GAR	23,658 ± 163 ^b^	8642 ± 95 b^c^	0.365 ± 0.000 ^b^	0.15 ± 0.00 ^a^	39.91 ± 0.51 ^c^
CAP	20,767 ± 690 ^cd^	7904 ± 185 ^cd^	0.380 ± 0.000 ^ab^	0.17 ± 0.00 ^a^	35.15 ± 0.09 ^d^
KHO	23,781 ± 924 ^ab^	8971 ± 175 ^ab^	0.377 ± 0.001 ^ab^	0.15 ± 0.00 ^a^	41.66 ± 1.55 ^c^
Control	14,201 ± 240 ^f^	5640 ± 143 ^f^	0.398 ± 0.013 ^ab^	0.09 ± 0.00 ^b^	54.91 ± 5.47 ^a^

* Mean values ± standard deviation; values with the same letter do not differ significantly from each other according to Tukey’s HSD test (*p* < 0.05). Abbreviations: GP (Giovanni Paolo); PP (Padre Pio); MON (Monlis); NOR (Norberto); GAR (Garfagnana); CAP (Cappelli); and KHO (Khorasan).

**Table 5:** Dough fermenting properties.

	Dough Development		Gas Release				
Samples *	Hm (mm)	T’2 (min)	H’m (mm)	T’1 (min)	V_-TOT_ (mL)	V_-REL_ (mL)	RC (%)
GP	26.05 ± 0.78 ^b^	38.15 ± 1.14 ^c^	76.40 ± 2.69 ^a^	62.15 ± 3.11 ^cd^	1839 ± 67 ^a^	559 ± 58 ^a^	70 ± 2 ^d^
PP	19.10 ± 0.14 ^c^	29.00 ± 2.50 ^d^	71.95 ± 2.76 ^ab^	114.45 ± 7.25 ^ab^	1580 ± 39 ^cde^	333 ± 52 ^cd^	79 ± 4 ^ab^
MON	20.75 ± 0.49 ^bc^	29.15 ± 1.04 ^d^	80.75 ± 0.64 ^a^	99.45 ± 24.24 ^abc^	1711 ± 11 ^b^	485 ± 8 ^ab^	72 ± 0 ^cd^
NOR	25.75 ± 1.91 ^b^	38.15 ± 1.04 ^c^	79.00 ± 1.98 ^a^	120.00 ± 4.15 ^ab^	1721 ± 2 ^ab^	503 ± 5 ^ab^	71 ± 0 ^d^
GAR	20.50 ± 0.71 ^bc^	34.30 ± 4.15 ^cd^	71.30 ± 4.53 ^ab^	81.45 ± 13.47 ^bcd^	1615 ± 27 ^bcd^	424 ± 21 ^bcd^	74 ± 1 ^abcd^
CAP	24.35 ± 2.05 ^bc^	54.00 ± 2.07 ^b^	64.90 ± 0.85 ^b^	129.45 ± 7.25 ^a^	1512 ± 26 ^de^	304 ± 22 ^d^	80 ± 2 ^a^
KHO	24.15 ± 2.76 ^bc^	30.45 ± 1.04 ^cd^	79.70 ± 3.82 ^a^	54.45 ± 5.18 ^d^	1639 ± 11 ^bc^	453 ± 27 ^abc^	73 ± 2 ^bcd^
Control	47.40 ± 0.69 ^a^	66.01 ± 3.11 ^a^	71.90 ± 2.56 ^ab^	85.01 ± 12.10 ^bcd^	1472 ± 21 ^e^	322 ± 21 ^d^	78 ± 2 ^abc^

* Mean values ± standard deviation, values with the same letter do not differ significantly from each other according to Tukey HSD test (*p* < 0.05). Abbreviations: GP (Giovanni Paolo); PP (Padre Pio); MON (Monlis); NOR (Norberto); GAR (Garfagnana); CAP (Cappelli); and KHO (Khorasan).

**Table 6:** Proximate chemical composition of the enriched breads (g 100 g^−1^ dm).

Samples *	Moisture	Protein	Ash	Fat	TDF
GP	37.84 ± 0.22 ^a^	19.6 ± 0.3 ^a^	4.18 ± 0.08 ^bc^	0.36 ± 0.01 ^c^	14.59 ± 0.05 ^b^
PP	36.57 ± 0.38 ^b^	19.2 ± 0.1 ^ab^	4.28 ± 0.05 ^b^	0.42 ± 0.01 ^b^	10.77 ± 0.15 ^d^
MON	37.11 ± 0.20 ^ab^	19.6 ± 0.2 ^a^	4.34 ± 0.10 ^b^	0.51 ± 0.02 ^a^	11.71 ± 0.60 ^cd^
NOR	37.20 ± 0.02 ^ab^	19.1 ± 0.1 ^ab^	4.63 ± 0.04 ^a^	0.44 ± 0.02 ^b^	15.08 ± 0.16 ^ab^
GAR	36.53 ± 0.03 ^b^	18.5 ± 0.1 ^c^	4.30 ± 0.03 ^b^	0.23 ± 0.01 ^d^	15.51 ± 0.07 ^ab^
CAP	36.31 ± 0.38 ^b^	18.9 ± 0.1 ^bc^	4.04 ± 0.06 ^c^	0.24 ± 0.01 ^d^	12.32 ± 0.25 ^c^
KHO	37.74 ± 0.16 ^a^	18.5 ± 0.1 ^c^	4.24 ± 0.03 ^b^	0.37 ± 0.01 ^c^	16.09 ± 0.10 ^a^
Control	34.86 ± 0.17 ^c^	15.4 ± 0.4 ^d^	3.44 ± 0.08 ^d^	0.07 ± 0.02 ^e^	10.75 ± 0.24 ^d^

* Mean values ± standard deviation, values with the same letter do not differ significantly from each other according to Tukey HSD test (*p* < 0.05). Abbreviations: GP (Giovanni Paolo); PP (Padre Pio); MON (Monlis); NOR (Norberto); GAR (Garfagnana); CAP (Cappelli); and KHO (Khorasan).

**Table 7:** Specific volume and textural properties of the studied functional breads.

Samples *	Specific Volume (mL g^−1^)	Hardness (N)	Chewiness
GP	2.46 ± 0.04 ^d^	17 ± 0.75 ^a^	12.77 ± 1.46 ^ab^
PP	2.47 ± 0.03 ^cd^	18.98 ± 1.33 ^a^	15.39 ± 1.2 ^a^
MON	2.6 ± 0.02 ^b^	14.19 ± 0.21 ^ab^	11.79 ± 0.08 ^ab^
NOR	2.55 ± 0.02 ^bcd^	15.63 ± 2.74 ^ab^	12.83 ± 1.75 ^ab^
GAR	2.57 ± 0.03 ^bc^	15.17 ± 2.11 ^ab^	12.21 ± 1.50 ^ab^
CAP	2.62 ± 0.02 ^b^	15.71 ± 0.01 ^ab^	13.09 ± 0.00 ^ab^
KHO	2.64 ± 0.01 ^b^	16.43 ± 0.39 ^ab^	13.66 ± 0.15 ^ab^
Control	2.97 ± 0.03 ^a^	11.44 ± 0.07 ^b^	9.99 ± 0.16 ^b^

* Mean values ± standard deviation, values with the same letter do not differ significantly from each other according to Tukey HSD test (*p* < 0.05). Abbreviations: GP (Giovanni Paolo); PP (Padre Pio); MON (Monlis); NOR (Norberto); GAR (Garfagnana); CAP (Cappelli); and KHO (Khorasan).

**Table 8:** Different color parameters of the studied functional breads.

	Crust Color			Crumb Color	
Samples *	*L*_crust_	*a*_crust_	*L*_crumb_	*a*_crumb_	*b*_crumb_
GP	56.11 ± 2.76 ^bc^	12.27 ± 0.80 ^ab^	60.19 ± 0.74 ^bc^	3.78 ± 0.02 ^b^	18.91 ± 0.09 ^b^
PP	54.28 ± 0.67 ^bc^	13.18 ± 0.36 ^a^	61.54 ± 1.31 ^bc^	5.27 ± 0.10 ^a^	19.37 ± 0.27 ^b^
MON	51. 69± 1.64 ^bc^	13.17 ± 0.65 ^a^	60.30 ± 0.59 ^bc^	4.84 ± 0.31 ^a^	21.33 ± 0.36 ^a^
NOR	51.34 ± 1.19 ^c^	13.33 ± 0.72 ^a^	59.30 ± 1.79 ^c^	5.22 ± 0.02 ^a^	21.56 ± 0.05 ^a^
GAR	55.47 ± 0.88 ^bc^	11.83 ± 0.39 ^ab^	60.26 ± 1.50 ^bc^	5.00 ± 0.11 ^a^	18.66 ± 0.05 ^b^
CAP	56.11 ± 1.37 ^bc^	12.34 ± 0.37 ^a^	62.79 ± 1.32 ^bc^	2.27 ± 0.57 ^c^	20.25 ± 0.33 ^ab^
KHO	57.6 ± 1.50 ^ab^	11.23 ± 0.74 ^ab^	64.04 ± 0.06 ^b^	2.17 ± 0.29 ^c^	20.30 ± 0.84 ^ab^
Control	62.50 ± 1.15 ^a^	10.02 ± 0.25 ^b^	69.90 ± 0.74 ^a^	- 0.57 ± 0.04 ^d^	14.82 ± 0.65 ^c^

* Mean values ± standard deviation, values with the same letter do not differ significantly from each other according to Tukey HSD test (*p* < 0.05). Abbreviations: GP (Giovanni Paolo); PP (Padre Pio); MON (Monlis); NOR (Norberto); GAR (Garfagnana); CAP (Cappelli); and KHO (Khorasan).

**Table 9:** Polyphenol fractions and antioxidant activity of the studied bran genotypes.

Samples *	Polyphenol Fractions (mg GAE 100 g^−1^ dm)				Antioxidant Activity (μmol TE g^−1^ dm)
	Soluble	Insoluble	IP/SP	Total	ABTS
GP	102 ± 2 ^d^	331 ± 12 ^bc^	3.24 ± 0.04 ^a^	433 ± 2 ^d^	73.0 ^b^
PP	125 ± 2 ^c^	326 ± 5 ^cd^	2.61 ± 0.05 ^b^	451 ± 9 ^c^	70.6 ^b^
MON	163 ± 2 ^b^	342 ± 7 ^a^	2.10 ± 0.01 ^d^	506 ± 3 ^b^	104.8 ^a^
NOR	178 ± 6 ^a^	339 ± 2 ^ab^	1.91 ± 0.08 ^e^	517 ± 9 ^a^	106.7 ^a^
GAR	124 ± 1 ^c^	301 ± 4 ^e^	2.42 ± 0.05 ^c^	426 ± 2 ^de^	58.3 ^c^
CAP	100 ± 4 ^d^	320 ± 4 ^d^	3.20 ± 0.03 ^a^	420 ± 5 ^e^	48.3 ^d^
KHO	92 ± 0 ^e^	291 ± 5 ^f^	3.18 ± 0.00 ^a^	382 ± 7 ^f^	45.5 ^e^

* Mean values ± standard deviation, values with the same letter do not differ significantly from each other according to Tukey HSD test (*p* < 0.05). Abbreviations: GP (Giovanni Paolo); PP (Padre Pio); MON (Monlis); NOR (Norberto); GAR (Garfagnana); CAP (Cappelli); and KHO (Khorasan).

**Table 10:** Polyphenol fractions and antioxidant activity of bran-fortified breads.

Samples *	Polyphenol Fractions (mg GAE 100 g^−1^ dm)				Antioxidant Activity (μmol TE g^−1^ dm)
	Soluble	Insoluble	IP/SP	Total	ABTS
GP	47 ± 2 ^c^	308 ± 7 ^d^	6.54 ± 0.2 ^c^	355 ± 6 ^c^	60.9 ^b^
PP	60 ± 2 ^b^	321 ± 12 ^bc^	5.38 ± 0.1 ^d^	380 ± 13 ^b^	47.9 ^f^
MON	65 ± 6 ^a^	332 ± 2 ^a^	5.08 ± 0.3 ^d^	398 ± 1 ^a^	51.1 ^e^
NOR	61 ± 4 ^b^	329 ± 4 ^ab^	5.40 ± 0.2 ^d^	390 ± 3 ^ab^	71.6 ^a^
GAR	47 ± 0 ^c^	307 ± 5 ^d^	6.48 ± 0.1 ^c^	354 ± 5 ^c^	58.4 ^c^
CAP	45 ± 1 ^c^	317 ± 4 ^cd^	6.97 ± 0.1 ^b^	362 ± 4 ^c^	56.7 ^d^
KHO	46 ± 2 ^c^	296 ± 5 ^e^	6.41 ± 0.0 ^c^	342 ± 5 ^d^	52 ^e^
Control	28 ± 2 ^d^	254 ± 5 ^f^	9.16 ± 0.5 ^a^	281 ± 5 ^e^	45.4 ^g^

* Mean values ± standard deviation, values with the same letter do not differ significantly from each other according to Tukey HSD test (*p* < 0.05). Abbreviations: GP (Giovanni Paolo); PP (Padre Pio); MON (Monlis); NOR (Norberto); GAR (Garfagnana); CAP (Cappelli); and KHO (Khorasan).

## Data Availability

The original contributions presented in the study are included in the article; further inquiries can be directed to the corresponding author.
